# An Unexpected Encounter With Miliary Tuberculosis in a Young Man With a Remote Exposure History

**DOI:** 10.7759/cureus.25454

**Published:** 2022-05-29

**Authors:** Abhishek Janardan, Malek Ayoub, Husna Khan, Pinky Jha, Valerie Carter

**Affiliations:** 1 Internal Medicine, Medical College of Wisconsin, Milwaukee, USA; 2 Internal Medicine, Medical College of Wisconsin, Wauwatosa, USA; 3 Dentistry, Midwestern University Chicago College of Osteopathic Medicine, Chicago, USA

**Keywords:** miliary tuberculosis, extrapulmonary manifestation, night sweats, immunocompetent adults, remote exposure history

## Abstract

Miliary tuberculosis (TB) represents a rare escalation of TB stemming from the hematogenous dissemination of *Mycobacterium tuberculosis.* Common extrapulmonary infection sites include the lymphatic system, musculoskeletal system, and central nervous system. The nonspecific motif of clinical symptoms involving joint pain, fever of unknown origin, and night sweats make the diagnosis of disseminated TB quite challenging. Long-term outcomes remain bleak. Untreated miliary TB is estimated to be fatal within one year. Here, we present a case of miliary TB in a 21-year-old male with a remote exposure history who was not immunocompromised.

## Introduction

Tuberculosis (TB) is a prominent global health concern due to its preventable mortality and morbidity. There has been a global decline in TB incidence and related deaths throughout the previous decades [1]. The 2021 World Health Organization Global Tuberculosis Report indicated that 10 million people contracted TB in 2020. Within the United States, the Centers for Disease Control and Prevention (CDC) reported 7,174 cases in 2020 [2]. Miliary TB is a unique form of disseminated TB. It presents in less than 2% of reported cases in immunocompetent individuals and up to 20% of extrapulmonary TB cases [3]. It impacts multiple organ systems and arises due to the progression of a primary infection or reactivation of a latent infection. Evidence of diffuse miliary infiltrates on chest radiograph or miliary tubercles within multiple organs is necessary to confirm the diagnosis of miliary TB. Some of the noteworthy manifestations include cold abscesses, spinal lesions, peripheral lymphadenopathy, and hepatosplenomegaly [3]. The gamut of clinical presentations of miliary TB constitutes a challenge for accurate and timely diagnosis. Here, we present the case of a young man with miliary TB with a remote exposure history.

## Case presentation

A 21-year-old Southeast Asian male with a medical history of anxiety, depression, and weight loss presented to his primary care provider due to an increased occurrence of lower lumbar pain over the previous month. The patient’s mother noted that he had felt fatigued for the past six months, only getting up to eat and use the restroom. In addition, the patient endorsed increasing amounts of lower back pain over the past month, reporting that his back felt “stiff and the pain was constant,” along with a soft, painless lump on his left buttock referred to as a “cyst/possible lipoma” by the patient. An X-ray of the lumbar spine and left hip was ordered. A lumbar X-ray displayed signs of scoliosis, and no lesions of the hip were noted. Labs displayed hemoglobin of 9.9 g/dL, hematocrit of 31%, red blood cell count of 3.87 × 10^6^/µL, and elevated ferritin levels. Further testing one month later revealed hemoglobin of 9.1 g/dL with normal mean corpuscular volume, ferritin of 703 µg/L, and elevated vitamin B12, lactate dehydrogenase, and haptoglobin. A benign hematology appointment was scheduled over the next month to analyze the root cause of the patient’s anemia.

**Figure 1:**
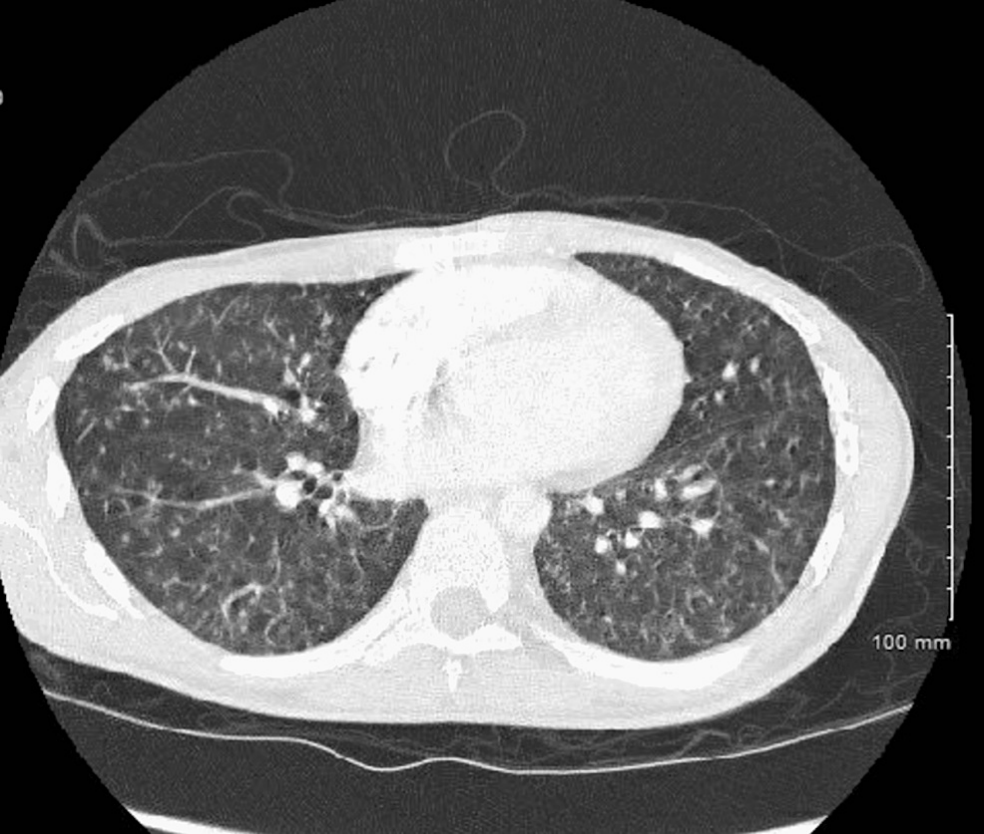
Miliary pulmonary nodules consistent with tuberculosis.

**Figure 2:**
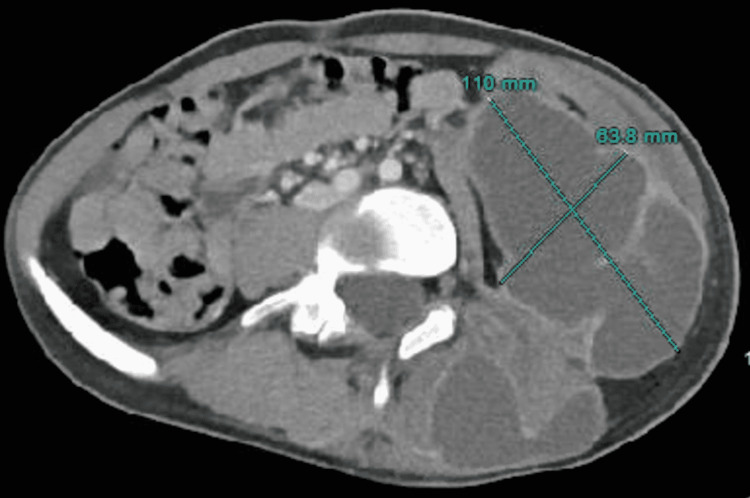
Collection centered within the left iliopsoas extending laterally into the abdominal oblique musculature and inferiorly into the inguinal region, measuring 110 mm × 63.8 mm.

A few weeks later, the patient’s mother contacted the Hematology-Oncology clinic with concerns about fevers and pains radiating from the swelling on the patient’s buttocks. The patient reiterated fatigue and hip and back pain over the previous two months, as well as intermittent fevers and night sweats over the last month, noting a constant need to change his t-shirt at night. He denied any chest pain, shortness of breath, or abdominal pain. Due to worsening hip and back pain with intermittent fever, a computed tomography (CT) scan was ordered. CT findings indicated multiple pulmonary nodules with a miliary pattern, loculated abscesses in the gluteus and ischium, erosive bony lesions of the left iliac and ischium, and calcified lymph nodes within the mediastinum. Further involvement of the left flank, retroperitoneum, and paraspinal region with accompanying lesions through the lungs were concerning for possible TB (Figures 1, 2).

The patient was then admitted to the hospital under isolation precautions. During a further interview, it was found that the patient’s father had a history of TB when the patient was two years old; associated treatment indicated that it was not drug-resistant TB. The father was given rifampin 600 mg, isoniazid 900 mg, pyrazinamide 3,500 mg, and ethambutol 3,200 mg for 2.5 months, followed by maintenance with rifampin and isoniazid for four months. Supplementation with pyridoxine was given, and treatment compliance was noted to be excellent. The patient himself also received prophylactic therapy during that time.

At admission, the blood differential was significant for a neutrophil composition of 88%, absolute neutrophil count of 10.33 × 10^3^/µL, and white blood cell count of 11.6 × 10^3^/µL. Treatment was initiated for osteomyelitis seen within the left iliac and left ischium bone erosions with involvement of the left sacroiliac joint. Empiric treatment with vancomycin 500 mg IV and cefepime was initiated per the recommendation of Infectious Disease consults. Quantiferon TB Gold Plus test was positive. Gluteal abscess culture displayed moderate acid-fast bacilli. Rifampin (450 mg), isoniazid (200 mg), ethambutol (800 mg), pyrazinamide (1000 mg), and pyridoxine B6 (50 mg) daily were initiated. Abdominal and paraspinal abscesses were deemed too viscous, and drainage was deferred. The patient was put in home isolation. Three weeks later, the patient had a virtual visit with Infectious Disease, indicating worsening left flank pain and abdominal abscesses requiring drainage. Aspirated samples of abscesses revealed the presence of *Mycobacterium tuberculosis*. The patient was recommended to continue antitubercular therapy for two more months, followed by 8-10 months of isoniazid and rifampicin. He was diagnosed with miliary TB. Upon returning for a follow-up three months later, the patient reported improvement in left-sided pain, improved ability to walk, return of appetite, and continued left flank drainage with about 20 ccs of output daily. Genitourinary ultrasound indicated reduced left lower quadrant fluid collection without any evidence of fistulization. Isoniazid (200 mg), rifampicin (450 mg), and B6 (50 mg) treatment was continued for a minimum of 12 months.

## Discussion

Miliary TB is characterized by the hematogenous seeding of *M. tuberculosis* from pulmonary or extrapulmonary focus to the capillary beds of highly vascular organs such as the liver, spleen, and bone marrow [4]. The nodules can be described as small, gray-to-brown-colored, round lesions and are called choroidal tubercles [3]. Clinically, it is seen as a subacute or chronic disorder with nonspecific symptoms such as fever, fatigue, and weight loss. Additional physical findings can include hepatosplenomegaly and lymphadenopathy. The disease has a bimodal age distribution, with the highest rates of infection occurring among young adults and the elderly [3]. The main modality of miliary TB seen in young adults is acute miliary TB.

Acute miliary TB commonly presents in individuals under 40 years of age [2]. Patients typically describe night sweats for one to two weeks, generalized constitutional symptoms of fever and fatigue, and organ-specific ailments. Cunha et al. reported spiking morning fever as a significant diagnostic feature. It is described as sweat engraving the patient’s silhouette on the bed, closely resembling a body’s shadow, and is commonly referred to as the “damp shadow sign” [5]. Other symptoms such as dry cough, scanty sputum, and dyspnea are also observed. Skin manifestations include erythematous macules or papules, known as TB malaria cutis [6]. Neurological manifestations, including TB meningitis, have been described in 30% of cases [7]. Musculoskeletal involvement occurs in 10% of cases, usually manifesting in the form of Pott’s spine. Symptoms of Pott’s spine include back pain, tenderness, paraparesis, and spinal curvature deformities [3].

Standard chemoprophylaxis for patients exposed to TB without progression to active infection involves a daily regimen of at least 5 mg/kg of isoniazid for nine months [8]. Isoniazid has been shown to reduce the risk of developing an active TB infection by 40-60%. Rifampin and pyrazinamide regimens have also yielded success as TB prophylaxis, but have been implicated with a greater side effect profile, especially in individuals unaffected with HIV. Malik et al. analyzed the impact of fluoroquinolone-based prevention therapy for TB and noted an effectiveness rate of 50-71% [9].

The standard antitubercular drug regimen for pulmonary TB also applies to miliary TB. The World Health Organization defines a standard TB regimen as a six-month regimen consisting of two months of intensive treatment involving rifampicin, isoniazid, pyrazinamide, and ethambutol, followed by a four-month continuation period with isoniazid and rifampicin [8]. Guidelines also suggest that treatment be extended to 12 months if TB meningitis is present or nine months if bone and joint TB are present [8]. However, there is no consensus on the most optimal treatment duration.

## Conclusions

Miliary TB can be difficult to diagnose due to the variety of initial clinical manifestations. Further, this is complicated by a lack of uniform guidelines to diagnose miliary TB. The diagnosis can perplex even the most experienced clinicians. General factors to consider for diagnosis include fever with a rise in evening temperature, night sweats lasting greater than six weeks that respond to antituberculosis treatment, pathological evidence of TB, and miliary pattern on chest radiograph.
